# Comprehensive Analysis to Identify *SPP1* as a Prognostic Biomarker in Cervical Cancer

**DOI:** 10.3389/fgene.2021.732822

**Published:** 2022-01-04

**Authors:** Kaidi Zhao, Zhou Ma, Wei Zhang

**Affiliations:** Department of Obstetrics and Gynecology, Zhongnan Hospital of Wuhan University, Wuhan, China

**Keywords:** SPP1, biomarker, cervical cancer, prognosis, immune infiltration

## Abstract

**Background:** *SPP1*, secreted phosphoprotein 1, is a member of the small integrin-binding ligand N-linked glycoprotein (SIBLING) family. Previous studies have proven *SPP1* overexpressed in a variety of cancers and can be identified as a prognostic factor, while no study has explored the function and carcinogenic mechanism of *SPP1* in cervical cancer.

**Methods:** We aimed to demonstrate the relationship between *SPP1* expression and pan-cancer using The Cancer Genome Atlas (TCGA) database. Next, we validated *SPP1* expression of cervical cancer in the Gene Expression Omnibus (GEO) database, including GSE7803, GSE63514, and GSE9750. The receiver operating characteristic (ROC) curve was used to evaluate the feasibility of *SPP1* as a differentiating factor by the area under curve (AUC) score. Cox regression and logistic regression were performed to evaluate factors associated with prognosis. The *SPP1*-binding protein network was built by the STRING tool. Enrichment analysis by the R package clusterProfiler was used to explore potential function of *SPP1*. The single-sample GSEA (ssGSEA) method from the R package GSVA and TIMER database were used to investigate the association between the immune infiltration level and *SPP1* expression in cervical cancer.

**Results:** Pan-cancer data analysis showed that *SPP1* expression was higher in most cancer types, including cervical cancer, and we got the same result in the GEO database. The ROC curve suggested that *SPP1* could be a potential diagnostic biomarker (AUC = 0.877). High *SPP1* expression was associated with poorer overall survival (OS) (*P* = 0.032). Further enrichment and immune infiltration analysis revealed that high *SPP1* expression was correlated with regulating the infiltration level of neutrophil cells and some immune cell types, including macrophage and DC.

**Conclusion:** *SPP1* expression was higher in cervical cancer tissues than in normal cervical epithelial tissues. It was significantly associated with poor prognosis and immune cell infiltration. Thus, *SPP1* may become a promising prognostic biomarker for cervical cancer patients.

## 1 Introduction

Cervical cancer remains the fourth most common cancer among women and accounts for 527,624 new diagnosed cases and 265,672 deaths in 2018 (Bray et al. (2018)). Cervical cancer continues to be the first or second leading cause of cancer-related death among women for many low- and middle-income countries (LMICs) (Wang et al. (2018)). Persistent HPV infection, especially types 16 and 18, is a high-risk factor but not the only one for cervical cancer (Revathidevi et al. (2020)). Host genetic factors may also be involved in tumor development. The major treatments for cervical cancer patients include surgery, chemotherapy, and radiotherapy. For patients with early-stage cervical cancer, 5-year survival is up to 91.5%, while the treatment of advanced cervical cancer is not ideal (Luan and Wang (2018)). The median survival time of metastatic cervical cancer patients is about 8–13 months, and the 5-year overall survival rate is only around 16.5% (Ferlay et al. (2013); van Meir et al. (2014)). Therefore, it is urgent to find more accurate biomarkers for early detection of cervical cancer and monitoring the disease progression.

Secreted phosphoprotein 1 (*SPP1*) is a secreted multifunctional phosphoprotein located in 4q13 with seven exons and six introns. *SPP1*, also known as osteopontin-like protein or early T-lymphocyte activation 1 protein, is a member of the small integrin-binding ligand N-linked glycoprotein (SIBLING) family which can specifically bind and activate matrix metalloproteinases (MMPs) in cancer (Su et al. (2020)). Its main biological functions are involved in immune response, biomineralization, and tissue remodeling. *SPP1* is also related to the growth, proliferation, migration, apoptosis, and chemotaxis of cells. Previous studies have proven that *SPP1* is overexpressed in a variety of cancers and can be used to predict the adverse consequences, including ovarian cancer (Zeng et al. (2018)), glioblastoma (Kijewska et al. (2017)), hepatocellular carcinoma (Wang et al. (2019)), and gastric cancer (Song et al. (2019)). Recently, the relationship between the expression of *SPP1* and chemotherapy resistance, such as prostate cancer and hepatocellular carcinoma, has also attracted the attention of researchers (Liu et al. (2016); Pang et al. (2019)), while no study has explored the correlation between *SPP1* and cervical cancer. Therefore, our study aimed to explore the expression of *SPP1* in cervical cancer tissues and its potential clinical values.

In our research, we utilized the cervical cancer RNA-seq data from The Cancer Genome Atlas (TCGA), Gene Expression Omnibus (GEO), and Genotype-Tissue Expression databases to compare the differential expression of *SPP1* between normal cervical tissues and cervical cancer samples. Next, we investigated the relationship between *SPP1* expression levels and clinical pathological features of cervical cancer. Furthermore, we explored the prognostic value of *SPP1* in cervical cancer. Besides, we performed gene enrichment analysis to reveal its potential functions. Finally, we analyzed the relationship between *SPP1* expression and immune infiltration and comprehensively explored its mechanism in inducing and promoting cervical cancer.

## 2 Materials and Methods

### 2.1 RNA Sequencing Data Collection and Analysis

To evaluate the *SPP1* expression level in pan-cancer, we downloaded data from the UCSC Xena (https://xenabrowser.net/datapages/). We selected samples from the TCGA database for the analysis of *SPP1* expression in tumor tissues, while the combined analysis of TCGA and Genotype-Tissue Expression (GTEx) databases was used for the normal tissue samples. GSE7803 (Platform: GPL96), GSE63514 (Platform: GPL570), and GSE9750 (Platform: GPL96) downloaded from GEO were used to obtain cervical cancer microarray data.

### 2.2 Correlation and Gene Set Enrichment Analysis

We used data collected from TCGA to perform correlation analysis between *SPP1* and other mRNAs in cervical cancer. To demonstrate the biological function of *SPP1*, we selected the top 100 genes most positively correlated with *SPP1* for enrichment analysis. EnrichGO function in the R package “clusterProfiler” was used to perform gene ontology (GO) enrichment, including BP, CC, and MF. Kyoto Encyclopedia of Genes and Genomes (KEGG) analysis was performed using the EnrichKEGG function of the R package “clusterProfiler.”

### 2.3 Survival Prognosis Analysis

We used the R package “survival” (version 3.6) to obtain the overall survival (OS) survival plots of *SPP1*. Selecting the cutoff value of 50% as the dividing threshold, the cohorts were divided into high-expression and low-expression groups. To evaluate the value of *SPP1* in predicting the prognosis of cervical cancer patients, we used the R package (version 3.6.3) “ROC” for analysis and “ggplot2” for visual.

### 2.4 Immune Cell Infiltration Analysis

We used the single-sample GSEA (ssGSEA) method from the R package GSVA (version 3.6) and Tumor Immune Estimation Resource (TIMER) database (http://timer.cistrome.org/) to comprehensively investigate molecular characterization of tumor–immune interactions in cervical cancer. In the literature, we examined the impact of *SPP1* expression on immune cell infiltration using gene expression profiling data. To investigate the correlation between *SPP1* expression and the abundances of tumor-infiltrating immune cells, *p*-values were calculated using the Wilcoxon rank-sum and Spearman’s rank correlation tests.

## 3 Results

### 3.1 The mRNA Expression Analysis of *SPP1* in Pan-Cancer

**FIGURE 1:**
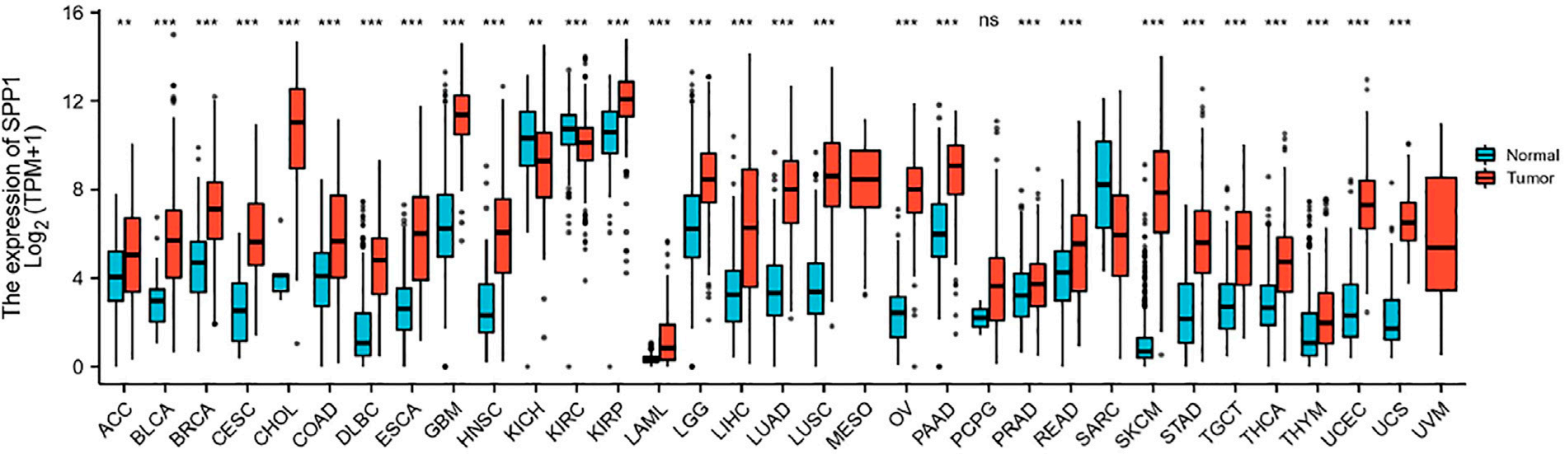
*SPP1* expression in normal and tumor tissues in TCGA and GTEx databases.

**FIGURE 2:**
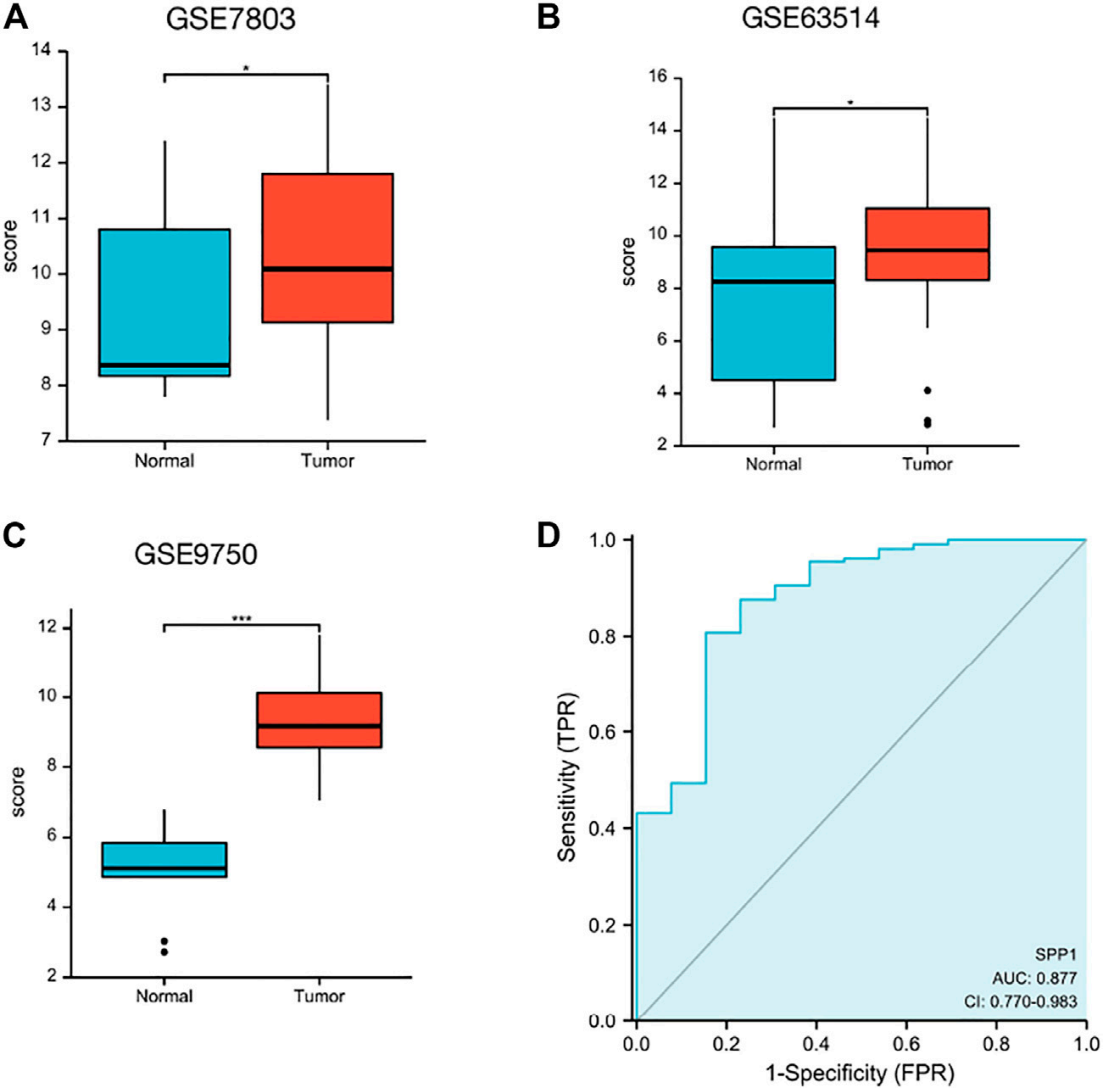
*SPP1* expression in the GEO database. **(A)** *SPP1* expression in normal and tumor tissues in cervical cancer from GSE7803. **(B)** *SPP1* expression in normal cervical epithelial and cervical cancer tissues from GSE63514. **(C)** *SPP1* expression in normal cervical tissues and cervical cancer epithelial component from GSE9750. **(D)** ROC curve of *SPP1* in cervical cancer. *X*-axis represents false-positive rates, and *Y*-axis represents true-positive rates.

Data downloaded from TCGA and GTEx were used to analyze *SPP1* expression in 33 types of cancer. The result revealed that *SPP1* was overexpressed in most cancers, including ACC, BLCA, BRCA, CESC, CHOL, COAD, DLBC, ESCA, GBM, HNSC, KIRP, LAML, LGG, LIHC, LUAD, LUSC, OV, PAAD, PRAD, READ, SKCM, STAD, TGCT, THCA, THYM, UCEC, and UCS. However, the expression of *SPP1* was low in KICH and KIRC (Figure 1). Furthermore, we assessed *SPP1* expression in cervical cancer in the GEO database, including GSE7803 (Platform: GPL96), GSE63514 (Platform: GPL570), and GSE9750, and the results confirmed that *SPP1* was overexpressed in cervical cancer tissues (Figures 2A–C). Additionally, we performed the receiver operating characteristic (ROC) curve to evaluate the feasibility of the *SPP1* expression level to distinguish cervical cancer tissues from normal cervical tissues. The area under the ROC curve (AUC) was 0.877, representing the quality of the test.

### 3.2 Clinical Relevance of the *SPP1* Expression in Cervical Cancer Patients

The characteristics of 306 primary cervical cancer patients with both clinical and gene expression data were downloaded from TCGA database. With the cutoff value of 50% as the dividing threshold, the patients were divided into a high–*SPP1* expression group (*n* = 153) and a low–*SPP1* expression group (*n* = 153). The correlation of the *SPP1* expression level and patients’ clinicopathologic characteristics was explored. We found that *SPP1* expression was significantly associated with T stage (*P* = 0.02), clinical stage (*P* = 0.02), and histologic type (*P* < 0.001) by using the chi-square test or Fisher’s exact test. The Wilcoxon rank-sum test revealed that *SPP1* expression was associated with age (*P* = 0.038) (Table 1).

**TABLE 1:** Correlation analyzed between *SPP1* expression and clinicopathologic characteristics in cervical cancer based on TCGA database.

Characteristic	Low expression of SPP1	High expression of SPP1	p value
N	153	153	
T stage, n (%)			0.020
T1	82 (33.7%)	58 (23.9%)	
T2	31 (12.8%)	41 (16.9%)	
T3	6 (2.5%)	15 (6.2%)	
T4	4 (1.6%)	6 (2.5%)	
N stage, n (%)			0.243
N0	73 (37.4%)	61 (31.3%)	
N1	27 (13.8%)	34 (17.4%)	
M stage, n (%)			0.699
M0	55 (43.3%)	61 (48%)	
M1	4 (3.1%)	7 (5.5%)	
Clinical stage, n (%)			0.020
Stage I	95 (31.8%)	67 (22.4%)	
Stage II	30 (10%)	39 (13%)	
Stage III	17 (5.7%)	29 (9.7%)	
Stage IV	9 (3%)	13 (4.3%)	
Radiation therapy, n (%)			0.726
No	63 (20.6%)	59 (19.3%)	
Yes	90 (29.4%)	94 (30.7%)	
Primary therapy outcome, n (%)			0.106
PD	7 (3.2%)	16 (7.3%)	
SD	2 (0.9%)	4 (1.8%)	
PR	4 (1.8%)	4 (1.8%)	
CR	101 (46.1%)	81 (37%)	
Race, n (%)			0.444
Asian	12 (4.6%)	8 (3.1%)	
Black or African American	13 (5%)	18 (6.9%)	
White	106 (40.6%)	104 (39.8%)	
Histologic type, n (%)			<0.001
Adenosquamous	40 (13.1%)	13 (4.2%)	
Squamous cell carcinoma	113 (36.9%)	140 (45.8%)	
Histologic grade, n (%)			0.954
G1	10 (3.6%)	9 (3.3%)	
G2	69 (25.2%)	66 (24.1%)	
G3	62 (22.6%)	57 (20.8%)	
G4	0 (0%)	1 (0.4%)	
Age (years), median (IQR)	45 (37, 54)	49 (40, 60)	0.038

We conducted the logistic regression method to further analyze the relationship between the *SPP1* expression level and the clinicopathologic characteristics of cervical cancer. The results showed that the expression level of *SPP1* was significantly associated with T stage (*P* = 0.004), clinical stage (*P* = 0.002), primary therapy outcome (*P* = 0.033), histologic type (*P* < 0.001), and age (*P* = 0.019) (Table 2).

**TABLE 2:** *SPP1* expression associated with clinicopathologic characteristics by logistic regression.

Characteristic	Total (N)	Odds ratio (OR)	*p* value
T stage (T2 and T3 and T4 vs. T1)	243	2.138 (1.278–3.609)	0.004
N stage (N1 vs. N0)	195	1.507 (0.821–2.786)	0.187
M stage (M1 vs. M0)	127	1.578 (0.451–6.294)	0.485
Clinical stage (Stage II and Stage III and Stage IV vs. Stage I)	299	2.051 (1.295–3.269)	0.002
Primary therapy outcome (SD and PR and CR vs. PD)	219	0.364 (0.135–0.893)	0.033
Histologic type (squamous cell carcinoma vs. adenosquamous)	306	3.812 (1.993–7.732)	<0.001
Age (>50 vs. ≤50 years)	306	1.743 (1.097–2.787)	0.019
Radiation therapy (yes vs. no)	306	1.115 (0.706–1.765)	0.641
Histologic grade (G2 and G3 and G4 vs. G1)	274	1.052 (0.411–2.731)	0.916

### Association Between *SPP1* Expression and Cancer Patient Survival Prognosis

**FIGURE 3:**
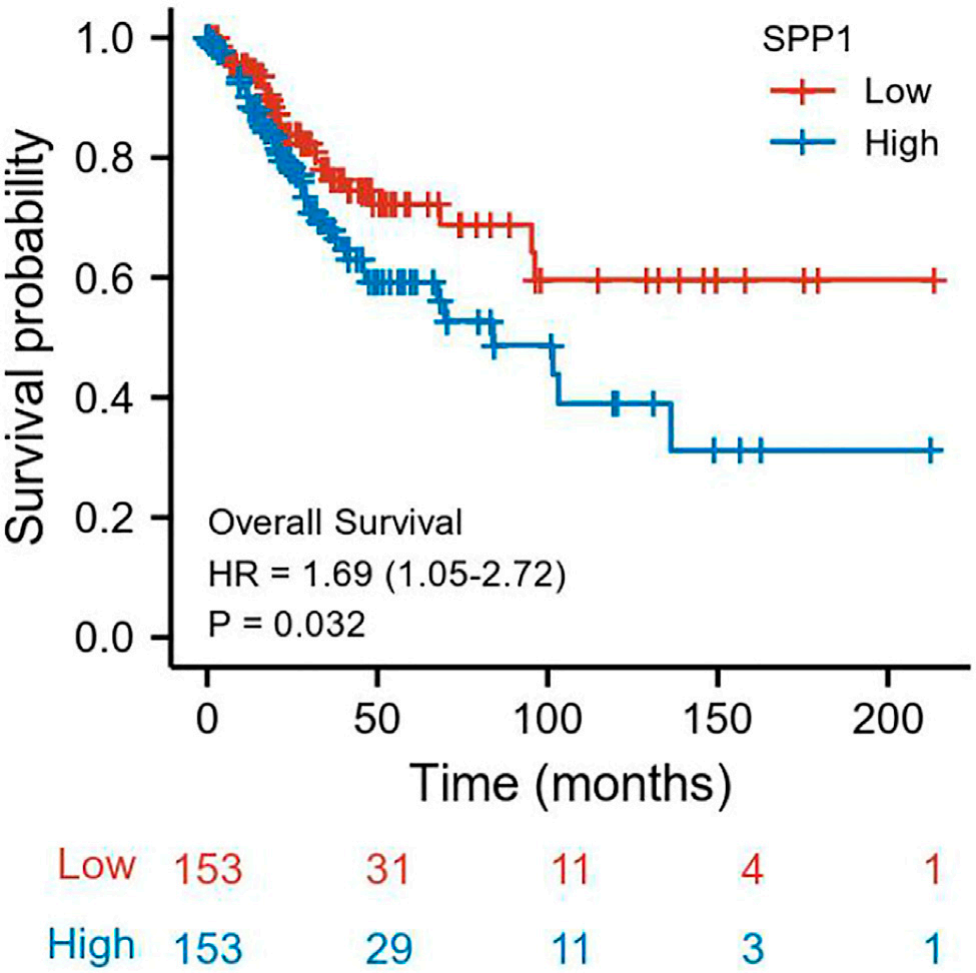
Association between *SPP1* expression and OS in cervical cancer patients.

We performed univariate and multivariate Cox analyses of overall survival (OS) in cervical cancer patients, and results are shown in Table 3. In univariate Cox analysis of *SPP1*, T stage (*P* = 0.025), N stage (*P* = 0.002), M stage (*P* = 0.023), and *SPP1* expression (*P* = 0.032) were associated with overall survival (OS) in cervical cancer patients. In the multivariate Cox model, we found that N stage (*P* = 0.002) and *SPP1* expression (*P* = 0.045) were still relevant to worse prognosis. Furthermore, we investigated the relationship between *SPP1* expression and overall survival (OS) of cervical cancer patients. According to the KM plot, patients with higher *SPP1* mRNA expression showed poorer prognosis than the lower group (HR = 1.69, 95% CI: 1.05–2.72, *P* = 0.032) (Figure 3). Thus, *SPP1* may become a promising prognostic biomarker for cervical cancer patients.

**TABLE 3:** Univariate and multivariate Cox analyses of prognostic factors in cervical cancer.

Characteristic	Total (N)	Univariate analysis		Multivariate analysis	
		Hazard ratio (95% CI)	*p* value	Hazard ratio (95% CI)	*p* value
T stage (T2 and T3 and T4 vs. T1)	243	1.906 (1.085–3.348)	**0.025**	1.193 (0.419–3.395)	0.741
N stage (N1 vs. N0)	195	2.844 (1.446–5.593)	**0.002**	3.117 (1.517–6.403)	**0.002**
M stage (M1 vs. M0)	127	3.555 (1.187–10.641)	**0.023**		
TP53 (high vs. low)	306	0.854 (0.537–1.356)	0.503		
Clinical stage (Stage II and Stage III and Stage IV vs. Stage I)	299	1.462 (0.920–2.324)	0.108	0.464 (0.160–1.345)	0.157
Radiation therapy (yes vs. no)	306	1.172 (0.694–1.981)	0.553		
Race (Black or African American and White vs. Asian)	261	1.537 (0.374–6.317)	0.552		
Age (>50 vs. ≤50 years)	306	1.289 (0.810–2.050)	0.284	0.658 (0.298–1.452)	0.299
Histologic type (squamous cell carcinoma vs. adenosquamous)	306	1.033 (0.543–1.969)	0.920		
Histologic grade (G2 and G3 vs. G1)	273	1.212 (0.378–3.882)	0.746		
SPP1 (high vs. low)	306	1.686 (1.046–2.719)	**0.032**	2.207 (1.019–4.777)	**0.045**

The value in bold indicates that p is less than 0.05, which is meaningful.

### 3.4 Correlation and *SPP1*-Related Gene Enrichment Analysis

**FIGURE 4:**
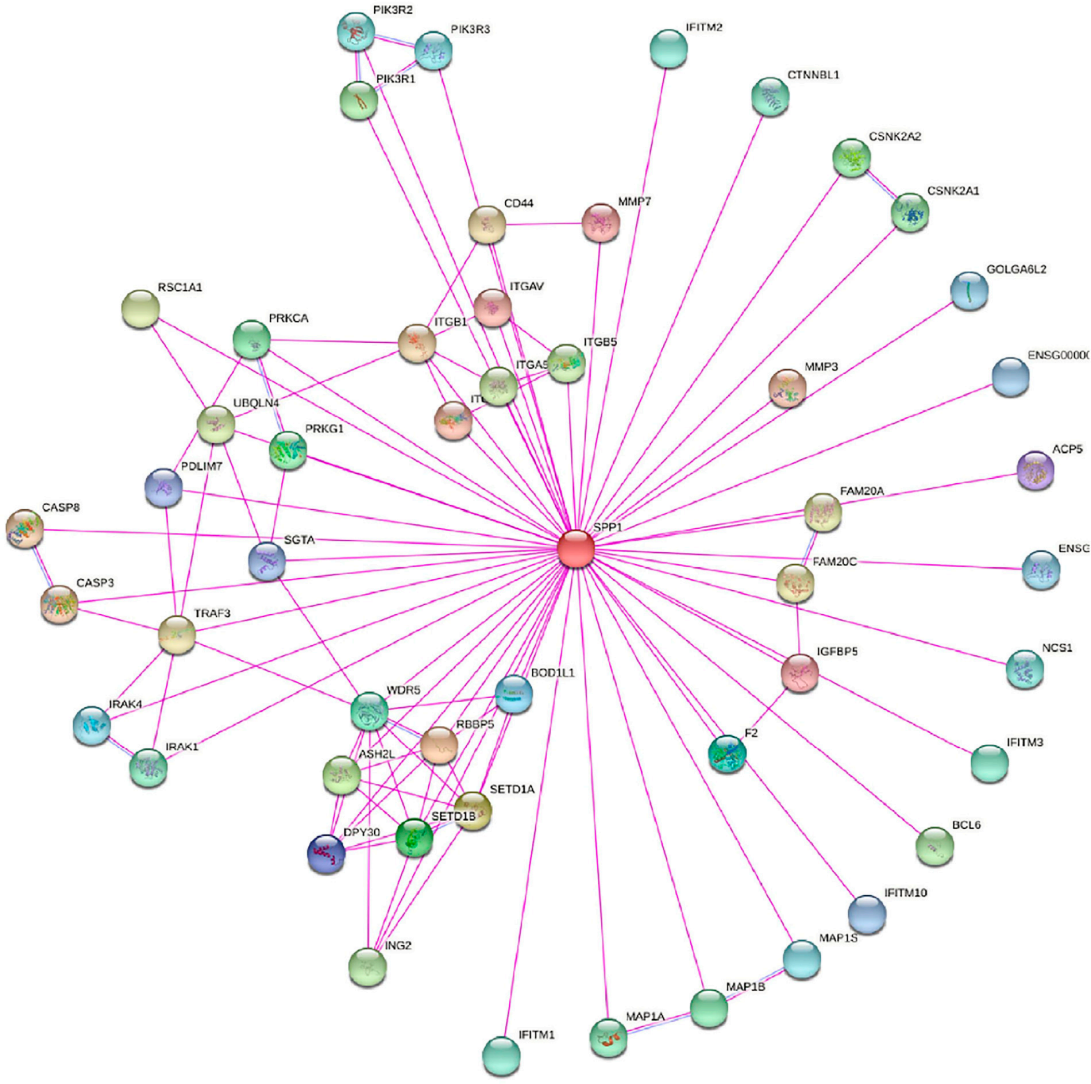
*SPP1*-binding proteins obtained by the STRING tool.

**FIGURE 5:**
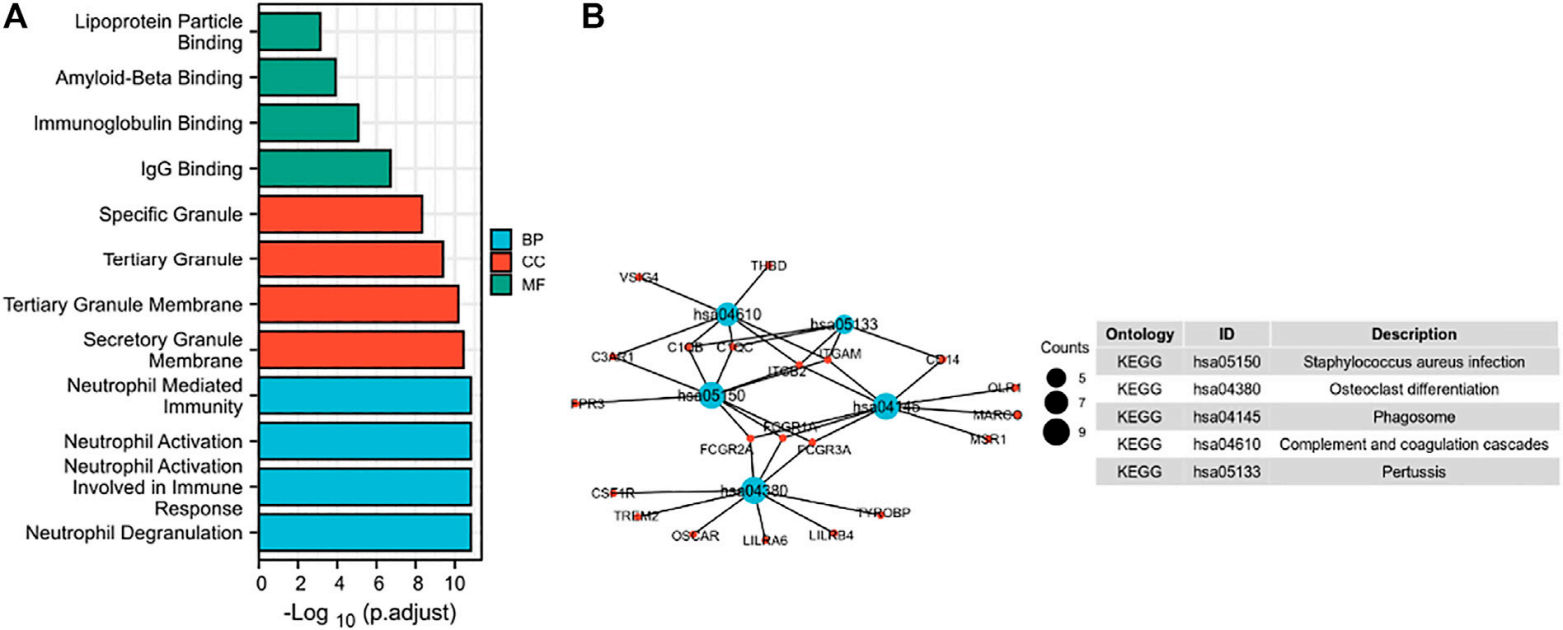
Function and pathway enrichment analysis of *SPP1* in cervical cancer. **(A)** Significant Gene Ontology terms (including BP, MF, and CC) of the top 100 genes most positively associated with *SPP1*. **(B)** Significant KEGG pathway of the top 100 genes most positively associated with *SPP1*.

In this study, we only considered physically binding protein interactions and obtained 50 experimental supported *SPP1*-binding proteins from the STRING network (Figure 4). We downloaded data from TCGA database to further investigate the function of *SPP1* and search *SPP1* expression–correlated genes for related pathway analysis. We obtained the top 100 most positively correlated genes with *SPP1* for GO and KEGG enrichment analysis by the “clusterProfile” R package. The GO analysis data showed that most of the genes were associated with neutrophil degranulation, neutrophil activation involved in immune response, neutrophil activation, and neutrophil-mediated immunity (Figure 5A). The KEGG data suggested that the “phagosome” may be related to the carcinogenic mechanism of *SPP1* (Figure 5B).

### 3.5 Relationship Between *SPP1* Expression and Immune Cell Infiltration

**FIGURE 6:**
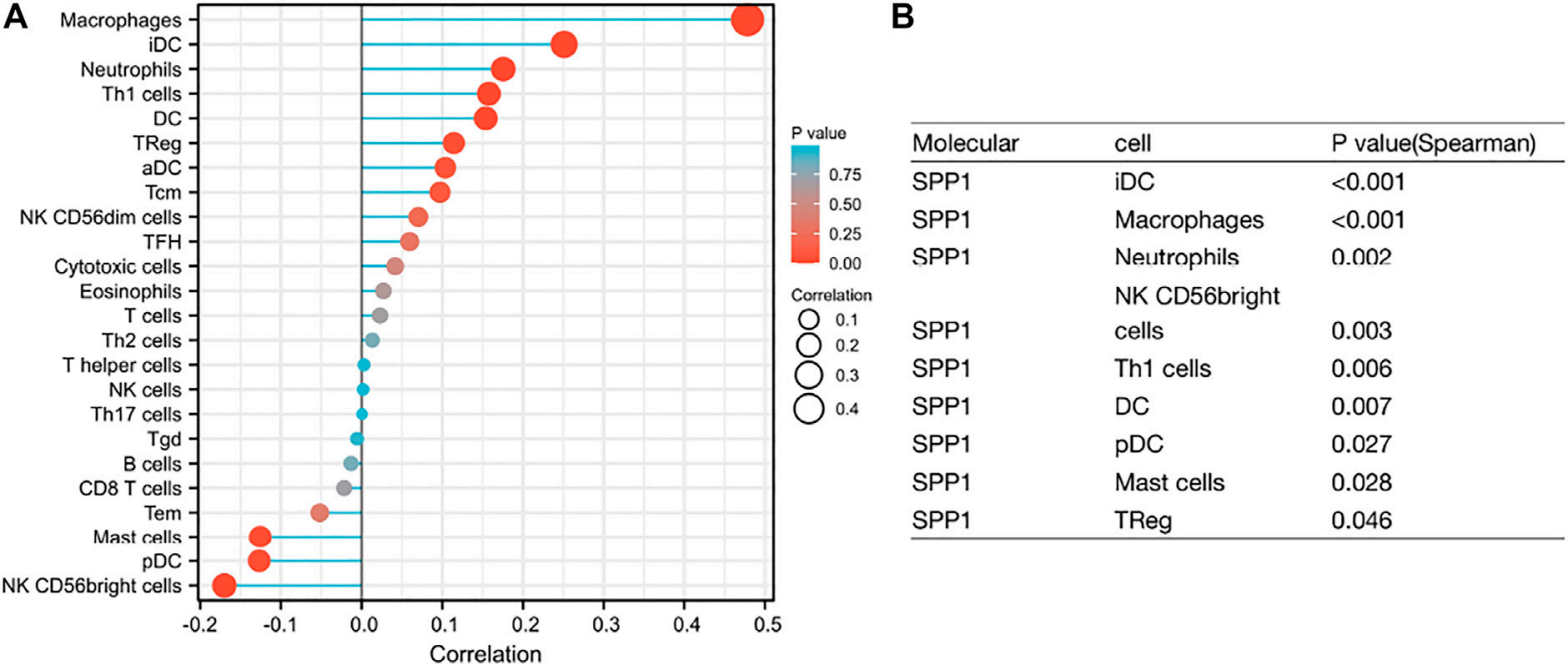
**(A)** Lollipop chart of *SPP1* expression level in 24 immune cells. **(B)** The immune cell infiltration associated with *SPP1* expression, P < 0.05, represents a significant result.

**FIGURE 7:**
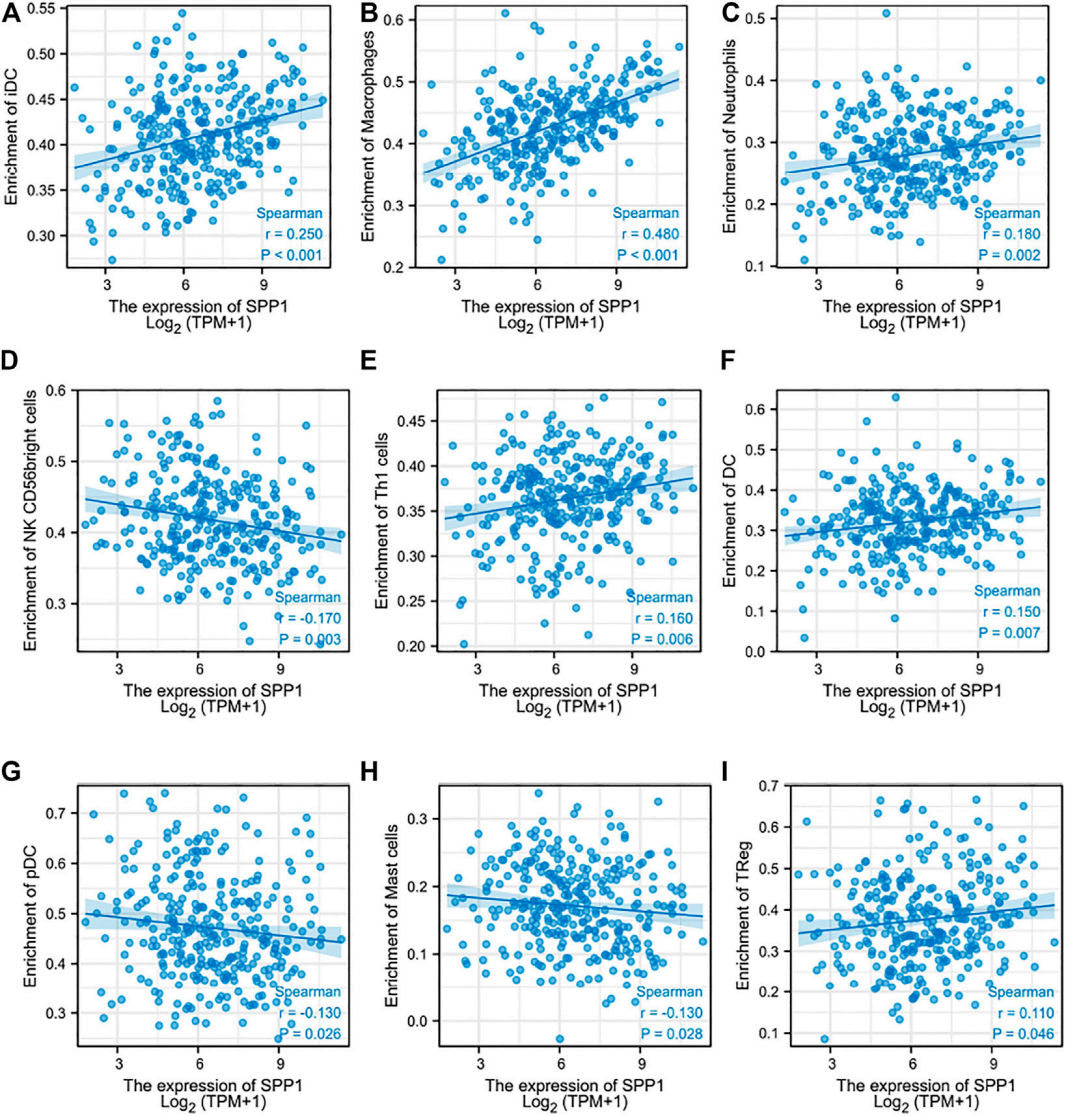
Correlation between *SPP1* expression and immune cell infiltration. **(A–I)** Correlation between *SPP1* expression and iDC, macrophages, neutrophils, NK CD56 bright cells, Th1 cells, DC, pDC, mast cells, and Treg cells.

**FIGURE 8:**
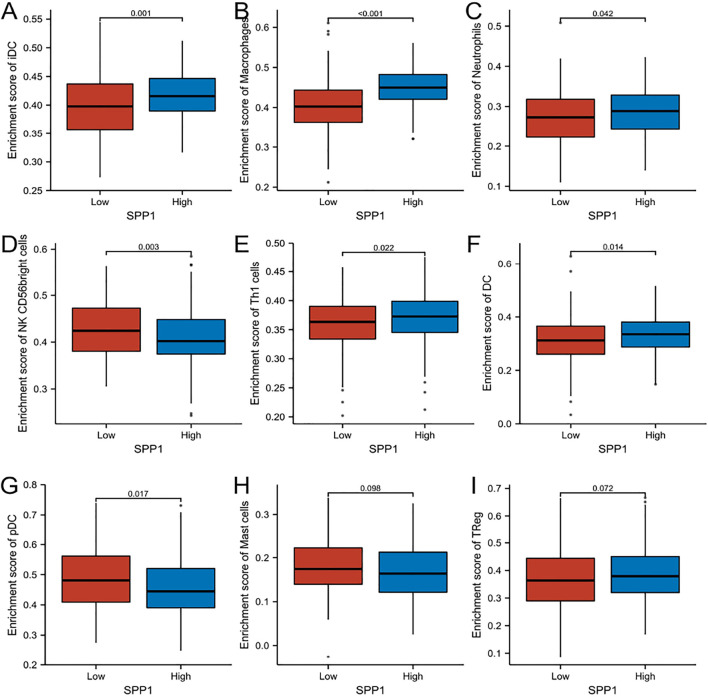
Comparison of immune cells between high– and low–*SPP1* expression groups. **(A–I)** Histogram showing the difference of iDC, macrophages, neutrophils, NK CD56 bright cells, Th1 cells, DC, pDC, mast cells, and Treg cell infiltration level between high–and low–*SPP1* expression groups.

**FIGURE 9:**
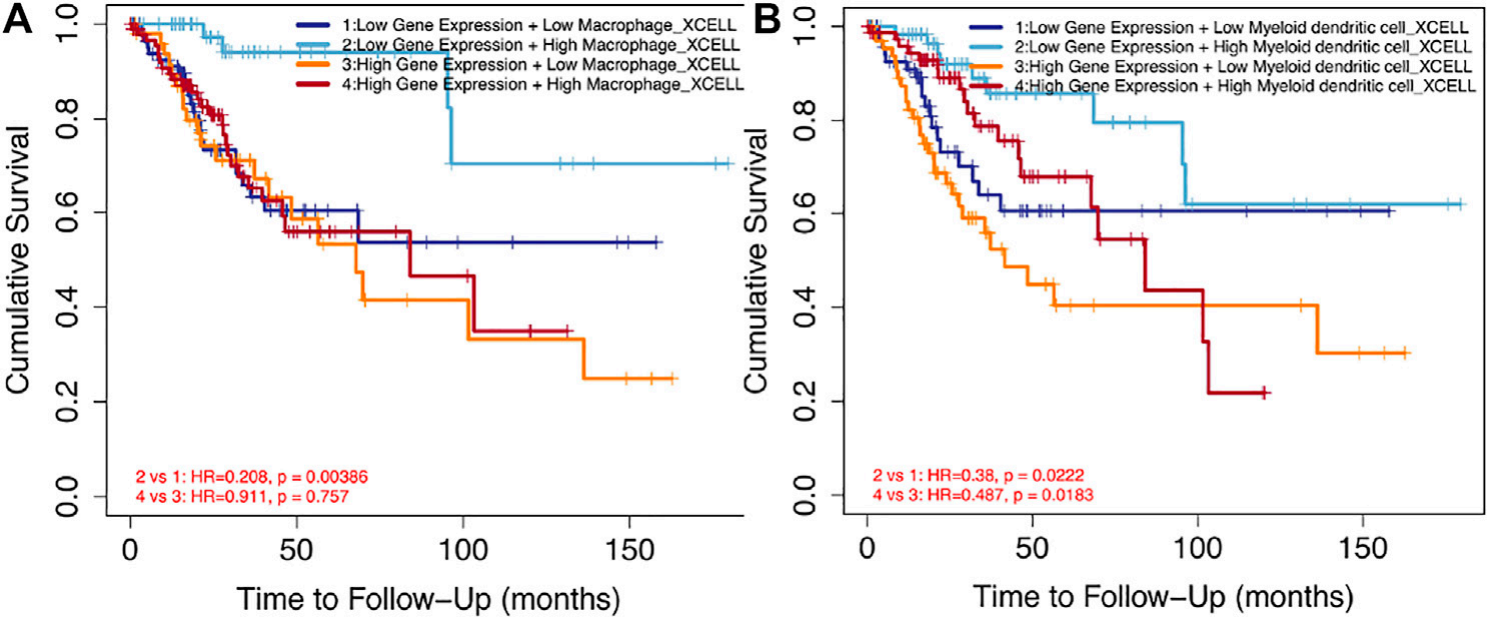
Impact of immune cell infiltration on prognosis in cervical cancer patients. **(A)** Clinical survival outcome of cervical cancer patients in the high-macrophage group. **(B)** Clinical survival outcome of cervical cancer patients in the high–DC cell group.

Through the previous enrichment analysis, we found that *SPP1* was mainly related to neutrophils and phagosomes. We hypothesized that there might be some relationship between *SPP1* and immune cells. Thus, we further assessed whether the *SPP1* expression level was associated with immune cell infiltration. We used ssGSEA from the R package with Spearman’s r to investigate the potential association between the *SPP1* expression level and 24 types of immune cells. The result revealed that *SPP1* expression had significant correlation with iDC, macrophages, neutrophils, NK CD56 bright cells, Th1 cells, DC, pDC, mast cells, and Treg cells (Figure 6). Further research showed that *SPP1* expression was positively correlated with infiltration levels of iDC (Figure 7A) (*r* = 0.250, *P* < 0.001), macrophages (Figure 7B) (*r* = 0.480, *P* < 0.001), neutrophils (Figure 7C) (*r* = 0.180, *P* = 0.002), Th1 cells (Figure 7E) (*r* = 0.160, *P* = 0.006), DC (Figure 7F) (*r* = 0.150, *P* = 0.007), and Treg cells (Figure 7I) (*r* = 0.110, *P* = 0.046). In contrast, *SPP1* expression was negatively correlated with that of NK CD56 bright cells (Figure 7D) (*r* = −0.170, *P* = 0.003), pDC (Figure 7G) (*r* = −0.130, *P* = 0.026) and mast cells (Figure 7H) (*r* = −0.130, *P* = 0.028). This prompted us to examine the relationship between the *SPP1* expression level and immune infiltration. Surprisingly, we found significant differences in infiltrating immune cell levels, including iDC, macrophages, neutrophils, NK CD56 bright cells, Th1 cells, DC, and pDC (*P* < 0.05), when *SPP1* expression was categorized into high and low groups (Figures 8A–G), while no significant difference in mast cells and Treg cells was noted (Figures 8H,I). Finally, we assessed the impact of immune cell infiltration on clinical survival outcome of cervical cancer patients by TIMER (http://timer.cistrome.org/). We found that high levels of macrophages and DC cells were associated with poor prognosis of cervical cancer patients (*P* < 0.05) (Figures 9A,B).

## 4 Discussion

Invasive cervical cancer remains the leading cause of cancer death among women worldwide (Shen et al. (2020)). Thus, it is necessary to find more accurate biomarkers to detect at an early stage and monitor disease progression. According to the previous studies, *SPP1* is overexpressed in various cancer types (Xu et al. (2017); Choe et al. (2018); Zhang et al. (2020)) and identified as a prognostic factor (Li et al. (2018); Chen J et al. (2019); Guo et al. (2020)), while to our knowledge, no study has explored the relationship of *SPP1* expression and cervical cancer. In our study, we attempted to explore the potential mechanism of *SPP1* in promoting cervical cancer and its feasibility as a molecular biomarker.

In pan-cancer analysis, we found that *SPP1* was upregulated in most cancer types. Further exploration revealed that higher *SPP1* expression was associated with reduced overall survival (OS) in cervical cancer patients. We performed logistic regression to evaluate the relationship between the *SPP1* expression level and the clinicopathologic characteristics of cervical cancer. The result showed that *SPP1* was significantly correlated with clinical stages. In addition, univariate and multivariate Cox analyses indicated that *SPP1* was an independent factor to predict prognosis of patients. All these aforementioned results and ROC analysis suggest that *SPP1* may be a promising prognostic biomarker for cervical cancer patients.

The tumor microenvironment (TME), composed of various types of immune cells, played an important role in tumor progression, metastasis, and treatment resistance (Usui et al. (2016)). The composition of tumor-infiltrating immune cells strongly influenced the tumor microenvironment and the behavior of the tumor. Our gene enrichment analysis revealed that the main biological function of *SPP1* was mainly involved in immune response. We next confirmed that *SPP1* expression correlated with immune cell infiltration. Hence, we hypothesized that *SPP1* may affect the tumor microenvironment by changing proportions of specific immune cell types, thereby promoting tumor progression and metastasis. It was, indeed, the case that *SPP1* had recently been shown to be an important component in maintaining the tumor microenvironment in AML (Ruvolo et al. (2019)). Our research demonstrated the significant positive correlation between macrophages and the expression of *SPP1*. Macrophages are important components of the tumor microenvironment, and tumor-associated macrophages play complex roles in cancer pathophysiology (Gibson et al. (2019)). A previous study found that *SPP1* was involved in the function, migration, and differentiation of macrophages (Zhang et al. (2017); Wei et al. (2019); Jaitin et al. (2019); Srirussamee et al. (2019)). A recent study also showed that *SPP1* was essential for M2-like macrophage, the tumor-associated macrophage, and promoted tumor growth (Chen P et al. (2019)). Furthermore, we found that the increased level of macrophages and DC infiltration were correlated with poor prognosis. Our results were supported by the findings of similar studies about this topic (Long et al. (2016); Ndiaye et al. (2019)). Certainly, the tumor microenvironment had a high level of complexity in its regulation; other immune cell types in the tumor microenvironment may also influence tumor cell survival, including iDC, neutrophils, NK CD56 bright cells, Th1 cells, DC, and pDC. Future studies were needed to further explore the relationship between *SPP1* expression and these cells.

In conclusion, we demonstrated that *SPP1* expression was upregulated in cervical cancer and significantly related to poor survival outcome. In addition to this, *SPP1* might participate in the occurrence and development of cervical cancer by influencing the infiltration level of immune cells. Therefore, our study revealed the role of *SPP1* in cervical cancer and identified a promising prognostic biomarker.

Although our study is the first work to explore the relationship between *SPP1* expression and cervical cancer, it also has some limitations. First, all of the data analyzed by bioinformatics methods in this study were downloaded directly from public databases, so it requires further validation by experimental investigations; second, the number of normal samples used as controls was considerably different from that of patients with tumor in the TCGA database; therefore, further studies based on an equal balance of sample size are necessary. Third, further validation studies with a long-term follow-up and larger cohorts of patients are needed to definitely validate *SPP1* as an OS predictor. Last but not least, our study laid the foundation for detailed studies of the correlation between *SPP1* and the tumor-associated immune microenvironment. However, more studies are required to explore the hypothesis in depth.

## Statement

The cervical cancer cell lines (Siha and Hela) present in this study were obtained from the Scientific Research Center of Zhongnan Hospital of Wuhan University. And normal cervical epithelial cell (END1) was donated by Wuhan University Basic Medical College.

## Data Availability

Publicly available datasets were analyzed in this study. These data can be found freely from TCGA data portal (https://portal.gdc.cancer.gov/) and GEO database (https://www.ncbi.nlm.nih.gov/geo/).

## Glossary

aDC: activated DC
ACC: adrenocortical carcinoma
BLCA: bladder urothelial carcinoma
BRCA: breast invasive carcinoma
CESC: cervical squamous cell carcinoma and endocervical adenocarcinoma
CHOL: cholangiocarcinoma
COAD: colon adenocarcinoma
DLBC: lymphoid neoplasm diffuse large B-cell lymphoma
ESCA: esophageal carcinoma
GBM: glioblastoma multiforme
GEO: Gene Expression Omnibus
GO: Gene Ontology
HNSC: head and neck squamous cell carcinoma
iDC: immature DC
KICH: kidney chromophobe
KIRC: kidney renal clear cell carcinoma
KIRP: kidney renal papillary cell carcinoma
KEGG: Kyoto Encyclopedia of Genes and Genomes
LAML: acute myeloid leukemia
LGG: lower grade glioma
LIHC: liver hepatocellular carcinoma
LUAD: lung adenocarcinoma
LUSC: lung squamous cell carcinoma
OS: overall survival
OV: ovarian serous cystadenocarcinoma
pDC: plasmacytoid DC
PAAD: pancreatic adenocarcinoma
PRAD: prostate adenocarcinoma
READ: rectum adenocarcinoma
SKCM: skin cutaneous melanoma
STAD: stomach adenocarcinoma
SPP1: secreted phosphoprotein 1
Tcm: T central memory
Tem: T effector memory
Tfh: T follicular helper
Tgd: T gamma delta.
TCGA: The Cancer Genome Atlas
TGCT: testicular germ cell tumor
THCA: thyroid carcinoma
THYM: thymoma
UCEC: uterine corpus endometrial carcinoma
UCS: uterine carcinosarcoma
